# Dickkopf-4 is frequently overexpressed in epithelial ovarian carcinoma and promotes tumor invasion

**DOI:** 10.1186/s12885-017-3407-1

**Published:** 2017-06-30

**Authors:** Shizhuo Wang, Heng Wei, Shulan Zhang

**Affiliations:** Department of Obstetrics and Gynecology, Shengjing Hospital, China Medical University, 36 San Hao Street, Heping District, Shenyang, Liaoning 110004 China

**Keywords:** Dickkopf-4, Epithelial ovarian carcinoma, Prognosis, Invasion

## Abstract

**Background:**

Dickkopf-4 (DKK4), a member of DKK family, appears to be a divergent protein. It remained multi-biological functions in carcinogenesis. The effect of DKK4 on the ovarian cancer cells remains unclear. This study detected the clinical significance of DKK4 in epithelial ovarian cancer (EOC) patients and its role in invasion.

**Methods:**

QRT-PCR and western blot analysis were used to examine the levels of DKK4 mRNA and protein in 33 EOC tissues and 33 benign ovarian tumors. Immunohistochemical analysis was performed to assess DKK4 expression in 239 EOC samples. siRNA-mediated DKK4 silence was conducted. Transwell assay was used to detect the invasive ability. Phalloidin was used to stain the formations of actin filaments.

**Results:**

The expressions of DKK4 mRNA and protein were elevated in EOC tissues as compared with those in benign ovarian tumors (*p* = 0.001 and <0.0001 respectively). Immunohistochemical results showed the strong expression of DKK4 protein was positively associated with late FIGO stage (*p* = 0.005) and poor disease free survival in univariate and multivariate analysis (*p* < 0.0001 and *p* = 0.001, respectively). SiRNA-mediated DKK4 knockdown inhibited cell invasive ability (all *p* < 0.0001) and the formations of actin filaments. DKK4 could promote the phosphration of c-JUN and JNK (*p* < 0.0001 and *p* = 0.001, respectively).

**Conclusions:**

Our results indicated that DKK4 might be contributed to predicting EOC progression and prognosis. DKK4 could promote the invasion of EOC through JNK activation.

## Background

Epithelial ovarian carcinoma is one of most common ovarian cancers. Its mechanism is unclear [1]. Its diagnosis and prognosis was late and poor due to its delayed symptoms and insensitive biomarkers [2, 3]. Consequently, it’s necessary for us to detect protein marker for predicting epithelial ovarian carcinoma progression and prognosis. The DKK family encodes secreted proteins in vertebrates (DKK1 to 4) [4–6]. DKK4 was one of DKK family member, it firstly acted as antagonist of Wnt proteins through binding to lipoprotein receptor-related protein 5/6 (LRP5/6), which induced the binding complex endocytosis and inhibiting Wnt/β-catenin activation [7–10]. Recently, DKK4 was found to be involved in carcinogenesis. Its functional role in tumor carcinogenesis was complicated. The earlier studies suggested that DKK4 was down-regulated in human tumors, for example in hepatocellular carcinoma and colorectal cancer, which indicated that DKK4 might act as a tumor suppressor by inhibiting Wnt/β-catenin signaling [11–14]. However, DKK4, induced by β-catenin activation, was found to be upregulated and promote invasion and angiogenesis in human colon cancers [15, 16]. DKK4 was co-overexpressed with MAPK3 and VAV3 in pancreatic ductal adenocarcinoma tissues [17]. DKK4 could also promote cell proliferation, invasion, and migration by activating the noncanonical c-Jun-NH2 kinase signaling pathway in renal cancer [18]. These studies suggest that DKK4 appears to be a divergent member of the DKK family, which remained multi-biological functions in carcinogenesis.

Till now, the expression and role of DKK4 in cancer invasion especially in epithelial ovarian cancer remained unclear. In the present study, we investigated the expression of DKK4 in epithelial ovarian carcinoma to prove the correlation between DKK4 with clinical parameters of EOC. Then we investigated its role in predicting EOC prognosis, regulating cancer cell invasion and its mechanism in regulating c-jun pathway.

## Methods

### Clinical samples

Frozen primary EOC tissues (*n* = 33), benign epithelial ovarian tumors (*n* = 33) and archival paraffin-embedded EOC samples (*n* = 239) were obtained from patients with primary epithelial ovarian tumors, aged 28 to 64 years, at Shengjing Hospital (Shenyang, China), from May 2009 to April 2014. Samples were selected for the study based on the criteria and followed up as previously reported [19]. After surgical treatment, the 239 ovarian carcinoma patients, whose archival paraffin-embedded samples were selected for immunohistochemical analysis, were followed up from May 1, 2009 to April 30, 2014. These 239 ovarian carcinoma patients were followed from the date of first resection surgery to the date of ovarian cancer recurrence or last observation. 99 patients were recurrent, 53 patients were lost to follow-up, and 87 patients were alive. Ethical approval for human subjects was obtained from the research ethics committee of ShengJing Hospital. Informed consent was obtained from patients enrolled in this study.

### Quantitative real-time RT-PCR

Tissue RNA and cell RNA, extracted from frozen paired tissues, were reverse transcribed using a TaKaRa RNA PCR kit. Quantitative real-time PCR was performed using the real-time PCR system 7300 (Applied Biosystems). PCRs were performed in triplicate. The relative levels of mRNA were analyzed by the 2 − ΔΔCt method. ΔCt = Ct (target)-Ct(β-actin). The mean expression level of DKK4 in normal tissues was used as control and considered as a value of 1.0, as described by Jarboe [20]. Using the following primers:

DKK4: 5′-TGGACTTCAACAACATCAGGAG-3′(forward)

DKK4: 5′-GGTATTGCAGTCCGTGTCAG-3′(reverse).

β-actin: 5′-CTTAGTTGCGTTACACCCTTTCTTG-3′(forward);

β-actin:5′-CTGTCACCTTCACCGTTCCAGTTT-3′ (reverse).

### Western blot

Tissue protein was extracted from frozen tissues and cells protein was lased with sodium dodecyl sulfate buffer. Proteins were extracted with RIPA buffer. Proteins with same concentration were separated on a 10% SDS-PAGE and then transferred to PVDF membranes. The membranes were blocked with 3% BSA in Tris-buffered saline with tween (TBST) and incubated with primary antibody DKK4 (1:500, R&D), anti-c-jun (1:400) (Abcam), anti-p-c-jun (1:350) (Santa Cruz), anti-JNK1/2 (1:400) (Abcam), anti-p-JNK (1:500) (Santa Cruz) followed by incubation with secondary antibody. Protein expression was visualized using enhanced chemiluminescence. Comparison between different groups were made by determining the specific protein/β-actin ratio of the immunoreactive area with densitometry. The tests were performed in triplicate.

### Immunohistochemistry analysis

Samples were fixed by formalin, embedded in paraffin and cut into sections (4–6 μm thick). Then the sections were deparaffnized, and stained using a streptavidin-biotin immunoperoxidase technique. The nucleus was stained with hematoxylin. DKK4 antibody (Abcam) was diluted as 1:250. Staining was scored as negative, weak, or strong, and rated by by multiplication of the intensity and positivity scores. (intensity: no staining, score 0; light yellow staining, score 1; moderate yellow staining, score 2; strong yellow staining, score 3; percentage < 5%, score 0; between 5% and 25%, score 1; between 26% and 50%, score 2; between 51% and 75%, score 3; >75%, score 4). Scores of 9–12 were considered as “strong”, scores of 5–8 were considered as “weak”, and scores of 0–4 were considered as “negative”, as mentioned by Hao et al. [21]. All of the stained sections were reviewed by two independent pathologists.

### siRNA transfection for DKK4 silence in vitro

37.5 × 10^4^ (6-well) HO-8910 and SKOV-3 cells were incubated in RPMI 1640 medium without antibiotics. After 12 h, 100 nmol/L (6-well) siRNA oligonucleotides was transfected using lipofectamine™ 2000 transfection reagent (Invitrogen). siRNA were designed: DKK4-specific siRNA (sense 5′-AGGAAGCAGAGA AACCCGGC-3′). Control cells were transfected with control siRNA.

### Transwell invasion assay

The transwell migration (Corning, USA) was performed using a chamber system with matrigel gel membrane (8.0 lm pore) from BD system (BD, USA). The 2 × 10^4^ cells were incubated into a 24-well plate with the upper chamber with 1% FBS medium, and the bottom was covered with the medium containing 20% FBS and 10 μg/ml of bovine fibronectin (chemoattractant) (Hyclone). The cells, migrated for 24 h, were fixed and counted in 10 high-powered (×200) fields under a microscope. The experiment was repeated three times.

### Drugs and reagents

To detect the ability of JNK on ovarian cancer cell invasion. 4 × 105 cells were pretreated with the JNK inhibitor SP600125 (0, 5 or 10 μM) in RPMI 1640 medium containing 5% FBS for 30 min before being added to the Matrigel-coated Transwell inserts. The cells, migrated for 12 h, were fixed and counted in 10 high-powered (×200) fields under a microscope as mentioned by Gonzalez-Villasana V et al. [22]. The experiment was repeated three times.

### Filamentous actin staining

Cells were washed with PBS (pH 7.4), fixed in methanol, rinsed and permeabilized with PBS containing 0.1% Triton X-100. Fixed cells were blocked for 1 h in 3% BSA and incubated with FITC-phalloidin (Sigma) for 30 min and then washed with PBS. The DNA dye DAPI (Molecular Probes) was used as nuclear stain. Images were obtained using a Laser confocal microscopy in 5 high-powered (×1000) fields. Multiple cells were categorized in each experimental point.

### Statistical analysis

Results from DKK4 expression, invasion ability, c-jun protein and JNK protein were evaluated by t-test. The Pearson chi-square and Fisher exact tests were used to examine DKK4 protein expression and its correlation with clinicopathological parameters. DFS was defined as the time from the date of first resection surgery to the date of ovarian cancer recurrence. Kaplan-Meier analysis was used for predicting DFS, assessed by the log-rank test. Hazard ratios and corresponding 95% confidence intervals were determined by Cox Regression model. Estimates of effect were estimated in models that included additional adjustment for clinical predictors, including DKK4, FIGO stage, age, cell differentiation, and lymph node metastasis. All statistical analyses were performed using SPSS 13.0. Statistical significance was defined as *p* < 0.05.

## Results

### DKK4 mRNA and protein were over-expressed in ovarian cancer

**Fig. 1:**
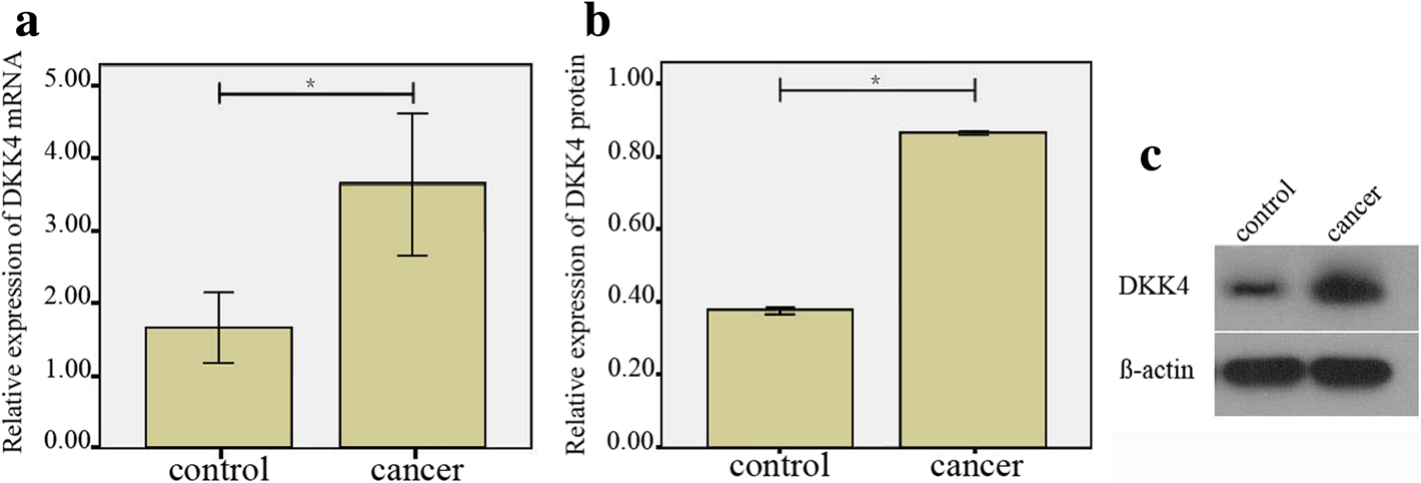
The expression of DKK4 mRNA and protein in EOC tissues. **a** qRT-PCR analysis of DKK4 mRNA levels in 33 cancer samples was up-regulated as compared with those in 33 benign ovarian tumors. **b** Western blot analysis of DKK4 protein levels in cancer samples was increased as compared with those in benign ovarian tumors. **c** Representative blots levels of DKK4 protein; *, *p* < 0.05

We compared the expression of DKK4 mRNA and protein in human EOC tissues and benign ovarian tumor tissues by qRT-PCR and western bolt. We found that the relative fold of DKK4 mRNA was significantly increased in EOC tissues (3.63 ± 2.84) than that in benign ovarian tumor tissues (1.66 ± 1.36) (*p* = 0.001; Fig. 1a). The relative level of DKK4 protein was significantly upregulated in EOC tissues (0.86 ± 0.01) than that in benign ovarian tumor tissues (0.37 ± 0.03) (*p* < 0.0001; Fig. 1b and c).

### The prognostic significance of DKK4 protein in epithelial ovarian patients

**Fig. 2:**
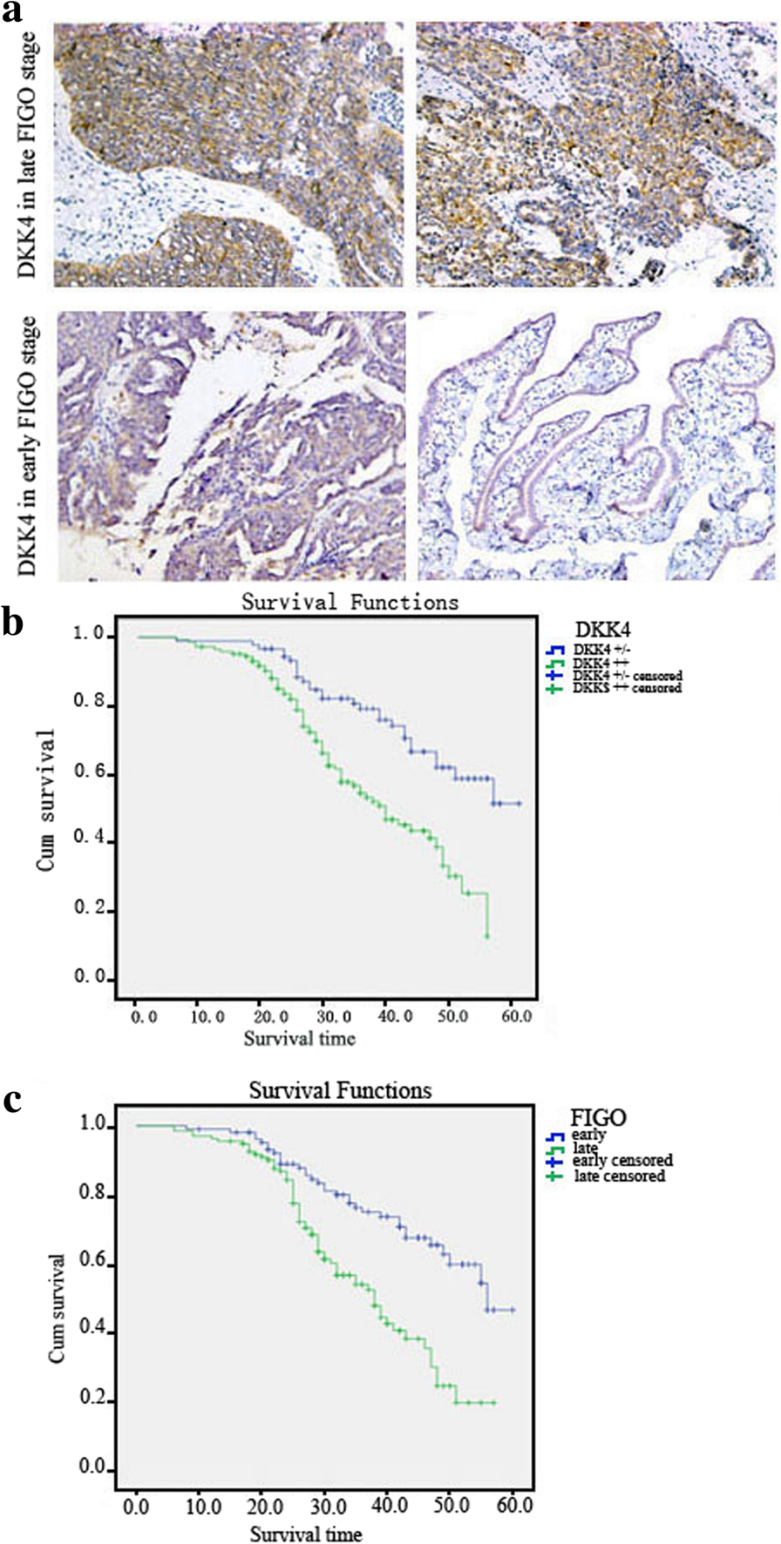
Expression and clinical significance of DKK4 protein expression in 239 EOC tissues. **a** Representative immunohistochemical expression of DKK4 protein in I-II and III-IV FIGO stage tissues. (100× magnification) (**b**) Kaplan–Meier curve showed the relationship between DKK4 expression and the Disease-free survival (DFS) of EOC patients. **c** The relationship between FIGO stage and the Disease-free survival (DFS) of EOC patients; *, *p* < 0.05

The result of immunohistochemistry analysis showed that DKK4 was positively expressed in epithelial ovarian cancer samples. DKK4 was strong expressed in 148/239 ovarian cancer samples, weak expressed in 72/239 ovarian cancer samples, while negative expressed in only 19/239 ovarian cancer samples. Meanwhile the strong expression of DKK4 protein in ovarian cancer samples was positively correlated with late FIGO stage with *p* = 0.005 (Fig. 2a, Table 1). The strong expression of DKK4 protein were not associated with age, cell differentiation or lymphatic metastasis in patients with epithelial ovarian cancer (all *p* > 0.05) (Table 1).

**Table 1:** Relationships between DKK4 and clinicopathological facotrs in 239 cases of EOC

	No.	DKK-4 expression			
		+/−	++	value	*P*
Age					
≤ 45	102	41	61	0.339	0.592
> 45	137	50	87		
FIGO stage					
I ~ II	105	51	54	8.750	0.005
III ~ IV	134	40	94		
Differentiation					
G1 ~ G2	111	46	65	0.996	0.351
G3	128	45	83		
Lymph metastasis					
no	168	61	107	0.748	0.387
yes	71	30	41		

The mean ± SD of the mean disease-free survival time for the entire group of 239 patients was 43.45 ± 1.18 (95% CI = 41.14–45.77) months. The mean disease-free survival time for patients with strong expression of DKK4 (38.32 ± 1.33 (95% CI = 35.73–40.92) months) was significantly lower as compared with that with weak or negative DKK4 expression (49.15 ± 1.67 (95% CI = 45.88–52.42) months, (*p* < 0.0001, log-rank test)) (Fig. 2b). The mean disease-free survival time for patients with late FIGO stage (37.78 ± 1.43 (95% CI = 34.97–40.58) months) was significantly lower as compared with that with early FIGO stage (48.70 ± 1.59 (95% CI = 45.58–51.82) months, (*p* < 0.0001, log-rank test)) (Fig. 2c). Unadjusted Cox regression revealed DKK4 level (HR = 2.10, 95% CI = 1.33–3.33, *P* = 0.001) and FIGO stage (HR = 2.18, 95% CI = 1.41–3.37, *P* < 0.0001) were independent disease-free prognostic factors for epithelial ovarian carcinoma patients. This association ((DKK4 level (HR = 2.18, 95% CI = 1.37–3.46, *P* = 0.001) and FIGO stage (HR = 2.21, 95% CI = 1.41–3.46, *P* = 0.001)) was also significant in the multivariate Cox model adjusted for age, cell differentiation, and lymph node metastasis. However, age (HR = 0.75, 95% CI = 0.50–1.13, *P* = 0.170), cell differentiation (HR = 0.80, 95% CI = 0.54–1.20, *P* = 0.29), and lymph node metastasis (HR = 1.25, 95% CI = 0.82–1.91, *P* = 0.30) were not significantly correlated with disease-free survival rates.

### DKK4 could promote ovarian cancer cell invasion

**Fig. 3:**
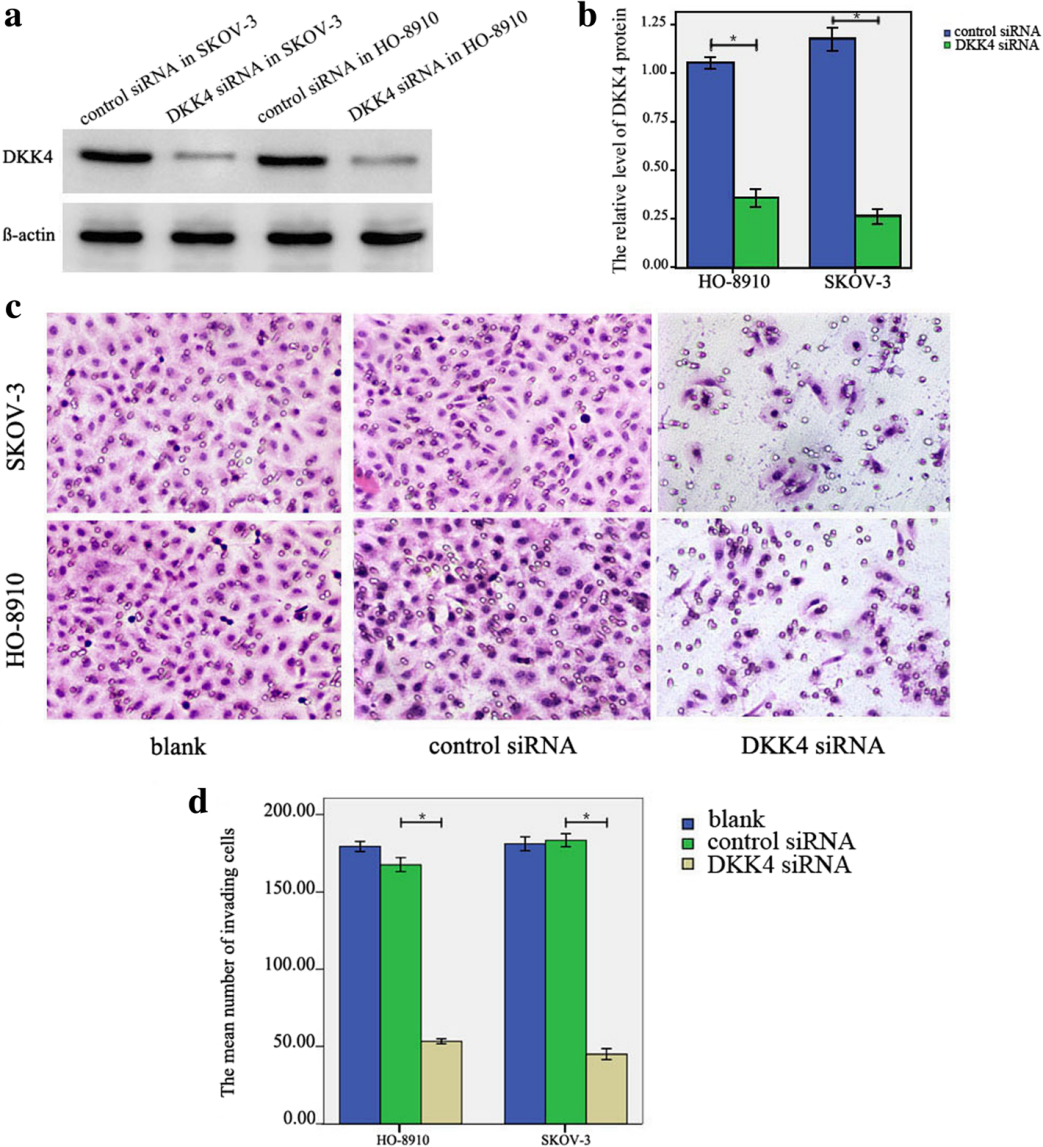
Transfection efficiency of siRNA mediated DKK4 knockdown and effect of DKK4 knockdown on cell invasion. **a**, **b** Western blot analysis of the knockdown efficiency of DKK4 siRNA in SKOV-3 and HO-8910 cells, *, *p* < 0.05. **c**, **d** Transwell assay showed that DKK4 knockdown inhibited the invasive ability of SKOV-3 and HO-8910 cell; *, *p* < 0.05; *N* = 3

DKK4 siRNA plasmid was transfected into SKOV-3 and HO-8910 cells lines, respectively. The knockdown efficiency of DKK4 protein in both SKOV-3 and HO-8910 cells were also confirmed by western blot (all *p* < 0.0001) (Fig. 3a and b). We detected the effect of DKK4 siRNA on ovarian cancer cell invasion (control siRNA vs. DKK4 siRNA) and the invasion ability of normal SKOV-3 and HO-8910 cells. Our results showed that DKK4 knockdown significantly decreased the incidence of invasion in ovarian cancer cells (SKOV-3: control siRNA (177.97 ± 29.59) vs. DKK4 siRNA (49.43 ± 23.57), *p* < 0.0001; HO-8910: control siRNA (167.63 ± 11.91) vs. DKK4 siRNA (53.23 ± 4.41), *p* < 0.0001) (Fig. 3c and d).

### DKK4 could promote the activity of JNK

**Fig. 4:**
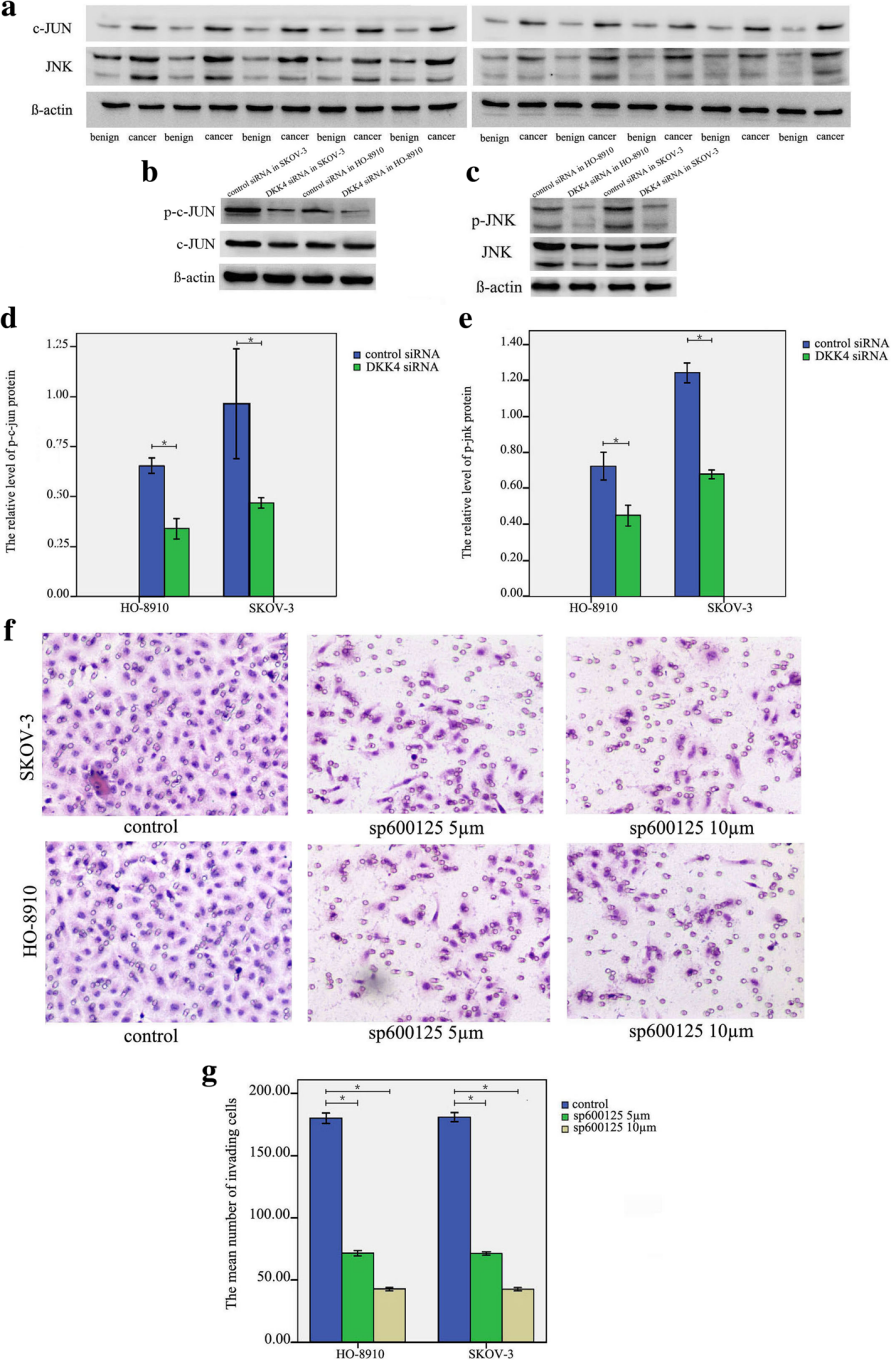
The analysis of DKK4 knowdown on cell invasion through inhibiting JNK activation. **a** Western blot analysis of c-JUN and JNK protein expression in 10 EOC cancer tissues and 10 benign ovarian tumors. **b**-**e** Western blot analysis of p-c-JUN and p-JNK level in DKK4 siRNA SKOV-3 and HO-8910 cells and control cells. **f**-**g** Transwell assay showed that JNK silence, mediated by JNK inhibitor SP600125, inhibited the invasive ability of SKOV-3 and HO-8910 cells; *, *p* < 0.05; *N* = 3

Previous studies found that the activation of JNK pathway could promote ovarian cancers progression [23, 24]. We detected the expression of JNK and c-JUN protein in 10 ovarian cancer tissues and 10 benign ovarian tumors. Our results showed that the levels of JNK and c-JUN protein in cancer tissues were both strong (Fig. 4a). Our results are consistent with previous studies [25, 26]. Then, we detected the activity of JNK and c-JUN in DKK4 siRNA cells. Our results showed that the phosphration of c-jun in DKK4 siRNA group was significantly decreased as compared with those in control siRNA group (DKK4- siRNA SKOV-3 group vs. control siRNA group, *p* = 0.001; DKK4- siRNA HO-8910 group vs. control siRNA group,*p* < 0.0001) (Fig. 4b and d). The phosphration of JNK in DKK4- siRNA group was significantly decreased as compared with those in siRNA control group (DKK4- siRNA SKOV-3 group vs. control siRNA group, *p* < 0.0001; DKK4- siRNA HO-8910 group vs. control siRNA group, *p* < 0.0001) (Fig. 4c and d). The band intensity of p-c-JUN or p-JNK was normalized to each corresponding band of c-JUN or JNK, respectively. The results indicated that DKK4 could promote JNK activation. Meanwhile, the inhibition of JNK activity, blocked by JNK specific inhibitor (SP600125), could decrease the invasive ability of ovarian cancer cells (SP600125 10 μM SKOV-3 group (42.43 ± 3.23) vs. control group (180.63 ± 9.67), *p* < 0.0001; SP600125 5 μM SKOV-3 group (70.97 ± 3.40) vs. control group (180.63 ± 9.67), *p* < 0.0001; SP600125 10 μM HO-8910 group (42.57 ± 3.56) vs. control group (179.83 ± 11.03), *p* < 0.0001;SP600125 5 μM HO-8910 group (71.17 ± 5.82) vs. control group (179.83 ± 11.03), *p* < 0.0001) (Fig. 4f and g). These results indicated that DKK4 could promote ovarian cancer cell invasion through promoting JNK activation.

### DKK4 could promote the formations of actin filaments

**Fig. 5:**
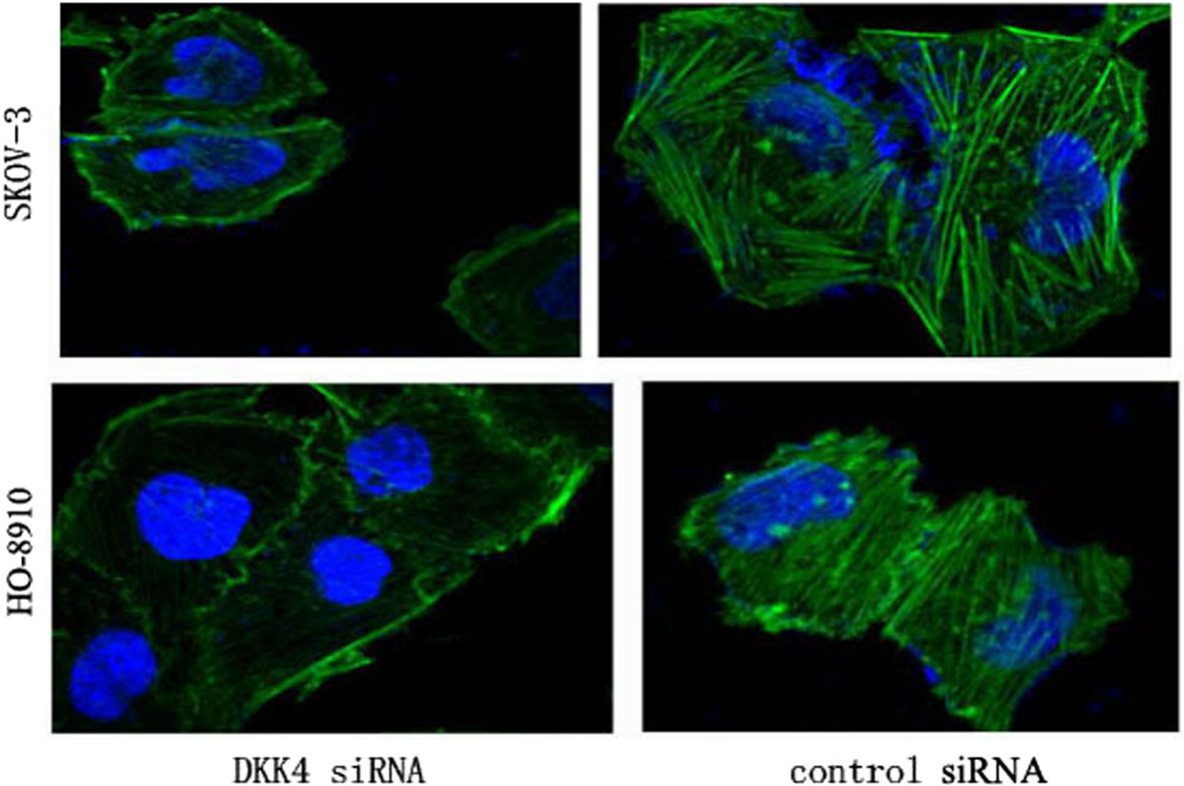
The effect of DKK4 knowdown on the formations of actin filaments in SKOV-3 and HO-8910 cells. The actin filaments in DKK4 siRNA groups were discontinuous, thinner, or even disappeared as compared with those in control groups

Many evidence indicated that actin filaments played an important role in promoting cell invasion [27, 28]. The activation of JNK pathway was known to be involved in modulating cytoskeleton like actin filaments [29, 30]. We examined the formation of actin by using phalloidin staining. Our results found that the majority of DKK4 silenced cells lost their actin filaments as compared with that in control siRNA groups (Fig. 5).

## Discussion

To date, DKK4 is the least studied and characterized member of the DKK family. DKK4 firstly, could act as a tumor suppressor by inhibiting the Wnt pathway [11–14]. However, DKK4 was later found that upregulated in human cancer, promoted tumor cell invasion and angiogenesis [15–18]. These results suggested that the role DKK4 in tumorigenesis was complex.

Till now, the expression pattern and mechanism of DKK4 in cancer was still obscure. In this study, our data supported a new role for DKK4 in human epithelial ovarian cancer. We for the first time investigated the expression of DKK4 and its function in EOCs. Our present results showed that DKK4 was upregulated at both the transcriptional and translational levels in EOCs. Immunohistochemistry analysis found that high DKK4 protein was associated with late FIGO stage, suggesting that DKK4 might be involved in EOC progression. A larger number of samples needed to be analyzed to testify our results. Although DKK4 overexpression was found in some cancers, like, colon [15, 16, 31], pancreatic [17], and renal cancer [18], limited information was available on the role of DKK4 protein in predicting cancer prognosis. In this report, our results showed that elevated DKK4 protein expression was correlated with poor prognosis for EOC patients. Meanwhile, DKK4 and FIGO stage were the independent predictors for EOC prognosis. Meanwhile, our in vitro assay also showed that DKK4 could promote EOC cell invasion.

The reason why DKK4 overexpression predicted poor prognosis for ovarian cancer patients and promoted invaion was unclear. Recently, Hirata H et al. also found that DKK4 could activate JNK pathway while inhibiting β-catenin signaling in renal cell carcinoma [18]. Ouyang et al. found that DKK4 might promote the development of pancreatic cancer through the abnormal activation of MAPK3 pathway [17]. In this report, our results found that DKK4 could promote c-jun and JNK protein phosphration, indicating DKK4 could promote invasion through activating JNK pathway. We also examine the expression of β-catenin and MAPK3 phosphration in DKK4 siRNA silenced cells, however, DKK4 failed to changed β-catenin or MAPK3 pathway (data not shown). The mechanism of DKK4 in activating JNK pathway was unclear. Hisham Bazzi et al. considered DKK4 overexpression as the constituted activation of Wnt/β-catenin signaling pathway [32, 33]. Wnt/β-catenin signaling pathway was activated in ovarian cancer progression [34]. Taken together, we hypothesized that DKK4 might be considered as a switch, shifting Wnt canonical to JNK signaling pathway. Large more studies were needed to testify the role of DKK4 in signal pathway activation.

The formation of actin filaments was one of the most important steps in promoting cell invasion [35]. The activation of JNK pathway was known to be involved in modulating cytoskeleton like actin filaments [36, 37]. Our results showed that DKK4 might promote the formations of actin filaments through activating JNK pathway. However, more studies also should be conducted to prove it.

## Conclusion

The present study observed that DKK4 mRNA and protein were elevated in EOC tissues. Immunohistochemical results showed the strong expression of DKK4 protein was positively associated with late FIGO stage and poor disease free survival time. SiRNA-mediated DKK4 knockdown inhibited cell invasive ability and the formations of actin filaments. DKK4 could promote the phosphration of c-JUN and JNK. In sum, we have shown that DKK4 was over-expressed, predicated poor prognosis and promoted tumor invasion through acitvating JNK in EOC carcinogenensis.

## Abbreviations

c-jun: c-Jun N-terminal kinase
DKK4: Dickkopf-4
EOC: epithelial ovarian cancer
MAP K3: Mitogen-activated protein kinase 3
p-c-jun: phosphrated c-Jun N-terminal kinase
TCF: T cell factor
